# Resistance of Animal Strains of *Pseudomonas aeruginosa* to Carbapenems

**DOI:** 10.3389/fmicb.2017.01847

**Published:** 2017-09-29

**Authors:** Marisa Haenni, Maxime Bour, Pierre Châtre, Jean-Yves Madec, Patrick Plésiat, Katy Jeannot

**Affiliations:** ^1^Unité Antibiorésistance et Virulence Bactériennes, ANSES-Université de Lyon Lyon, France; ^2^Centre National de Référence de la Résistance aux Antibiotiques, Centre Hospitalier Universitaire de Besançon Besançon, France

**Keywords:** *P. aeruginosa*, carbapenems, efflux pump, OprD porin, MexAB-OprM, MexXY/OprM, veterinary strains

## Abstract

Carbapenems are major antibiotics reserved to human medicine. This study aimed to investigate the mechanisms of carbapenem resistance of a selection of *Pseudomonas aeruginosa* veterinary strains from the French network Resapath. Thirty (5.7%) imipenem and/or meropenem non-susceptible *P. aeruginosa* of canine (*n* = 24), feline (*n* = 5), or bovine (*n* = 1) origin were identified in a large collection of 527 veterinary strains gathered by the Resapath. These resistant isolates belonged to 25 MultiLocus Sequence Types (MLST), of which 17 (68%) are shared with clinical (human) strains, such as high risk clones ST233 and ST395. Interestingly, none of the veterinary strains produced a carbapenemase, and only six of them (20%) harbored deletions or insertion sequence (IS) disrupting the porin OprD gene. The remaining 24 strains contained mutations or IS in various loci resulting in down-regulation of gene *oprD* coupled with upregulation of efflux system CzcCBA (*n* = 3; activation of sensor kinase CzcS ± CopS), MexEF-OprN (*n* = 4; alteration of oxido reductase MexS), MexXY (*n* = 8; activation of two-component system ParRS), or MexAB-OprM (*n* = 12; alteration of regulator MexR, NalC ± NalD). Two efflux pumps were co-produced simultaneously in three mutants. Finally, in 11 out of 12 strains displaying an intact porin OprD, derepression of MexAB-OprM accounted for a decreased susceptibility to meropenem relative to imipenem. Though not treated by carbapenems, animals thus represent a reservoir of multidrug resistant *P. aeruginosa* strains potentially able to contaminate fragile outpatients.

## Introduction

*Pseudomonas aeruginosa* is a major human pathogen. However, its role in animal infections as well as the resistance mechanisms developed by veterinary strains to treatments have rarely been studied (Cole et al., 1998). As most of the antipseudomonal antibiotics are strictly reserved to human medicine to preserve their activity, chemotherapy of infected animals relies on a few aminoglycosides, fluoroquinolones, and polymyxins, which are most often dispensed under the form of topical preparations (EFSA, 2013; WHO, 2016). Last-line antibiotics such as carbapenems (e.g., imipenem, meropenem, doripenem) are not expected to be used in this context and thus to select resistant strains. In contrast, the large consumption of carbapenems in hospitals worldwide exerts a strong selective pressure on clinical (human) populations of *P. aeruginosa*, promoting the emergence of resistant clones (Sievert et al., 2013). These resistant bacteria produce horizontally-acquired, carbapenem-hydrolyzing ß-lactamases (i.e., carbapenemases) belonging to Ambler's class A, B, or D, or/and are deficient in porin OprD, the main route of diffusion of carbapenems across the outer membrane in *P. aeruginosa* (Lister et al., 2009; Rodriguez-Martinez et al., 2009; Sanbongi et al., 2009; Riera et al., 2011; Ocampo-Sosa et al., 2012; Fournier et al., 2013b). Data gleaned over the years have shown that the alteration of the OprD uptake pathway may be caused by a variety of genetic events that disrupt the *oprD* gene, down-regulate its expression, or introduce deleterious amino acid changes in the porin sequence. Additionally, expression of *oprD* and operons coding for RND (Resistance Nodulation cell Division) pumps such as MexXY, CzcCBA, and MexEF-OprN can be inversely co-regulated in some single-step mutants encountered in the clinical setting (Poole, 2005; Lister et al., 2009; Muller et al., 2011; Fournier et al., 2013b; Li et al., 2015; Richardot et al., 2016). These mutants exhibit a cross resistance to carbapenems and various pump substrates by the conjunction of both membrane impermeability (OprD loss) and efflux mechanisms. It should be mentioned here that the imipenem molecule itself is not exported by any of the efflux systems of *P. aeruginosa* while meropenem and doripenem are substrates for MexAB-OprM (Maseda et al., 2000; Masuda et al., 2000; Okamoto et al., 2002). In so called *agrW2* mutants, the mutational activation of a two-component system, ParRS, results in reduced *oprD* transcription and concomitant upregulation of two operons, *mexXY* and *arnBCADTEF-ugd*, this latter promoting the addition of 4-aminoarabinose to lipid A of lipopolysaccharide (Muller et al., 2011). As a result, these mutants display an increased resistance to carbapenems, aminoglycosides, cefepime, fluoroquinolones, and polymyxins (Fournier et al., 2013b; Guénard et al., 2014). Mutational activation of another two-component phospho-relay, CzcRS, also leads to higher carbapenem resistance by repression of gene *oprD* expression, and to a stronger efflux of metal ions such as Zn^2+^ through the pump CzcCBA (Perron et al., 2004). Finally, alteration of a gene encoding a putative oxidoreductase, named MexS, can again be source of multidrug resistance in clinical strains of *P. aeruginosa* (Riera et al., 2011; Fournier et al., 2013b; Richardot et al., 2016). In this third type of mutants, dubbed *nfxC*, impairment of MexS enzymatic activity results in activation of a LysR-type transcriptional regulator, MexT, which in turn triggers expression of operon *mexEF-oprN* while repressing those of gene *oprD* (Köhler et al., 1999; Sobel et al., 2005b). The phenotype associated with *nfxC* mutations is characterized by a decreased susceptibility to carbapenems, fluoroquinolones, chloramphenicol, and trimethoprim. As mentioned above, MICs of meropenem and doripenem are dependent upon the expression levels of *mexAB-oprM*, and thereby can be influenced by mutations in genes such as *mexR, nalC*, and/or *nalD*, that are directly or indirectly involved in the regulation of this operon (Cao et al., 2004; Llanes et al., 2004; Sobel et al., 2005a). In the corresponding mutants *nalB, nalC*, and *nalD*, MICs of fluoroquinolones, macrolides, tetracyclines, chloramphenicol, and all the ß-lactams except imipenem are increased 2- to 16-fold (Masuda et al., 2000).

Since the first description of an IMP-1 expressing *P. aeruginosa* in Japan in the 1990s, the number of carbapenemase types and the number of clinical *P. aeruginosa* strains producing them has never ceased to increase worldwide (Potron et al., 2015). Similar strains have occasionally been recovered from animals (Al Bayssari et al., 2015).

A better understanding of the epidemiological and mechanistic aspects of carbapenem resistance in veterinary strains is obviously needed to anticipate and prevent subsequent therapeutic issues in humans. This study shows that the resistance to carbapenems in animal strains of *P. aeruginosa* is underestimated, and relies on non-enzymatic mechanisms likely selected by veterinary drugs. However, the presence of high-risk clones in both humans and animals highlights potential cross-contaminations between these two populations.

## Materials and methods

### Bacterial strains

A total of 30 non-duplicate veterinary strains of *P. aeruginosa* showing non-susceptibility to imipenem and/or meropenem according to the current Clinical and Laboratory Standards Institute breakpoints (CLSI, MIC >2 mg/L), were included in this study. These strains were collected between 2009 and 2014 by the French bacterial resistance surveillance network Resapath (http://www.resapath.anses.fr), from dogs (*n* = 24), cats (*n* = 5), and a bovine (*n* = 1). Wild-type reference strain PAO1 and its derived mutants PT629, PAO7H, and CMZ091 which overproduce efflux pumps MexAB-OprM, MexEF-OprN, and MexXY/OprM, respectively were used as controls in RT-qPCR experiments (Köhler et al., 1997a,b; Muller et al., 2011).

### Susceptibility testing and screening of carbapenemase-producing strains

The minimal inhibitory concentrations (MICs) of selected antibiotics were determined by the conventional microdilution method in Mueller-Hinton broth (MHB, Biorad) containing adjusted concentrations of Ca^2+^ (from 20 to 25 mg/L) and Mg^2+^ (from 10 to 12.5 mg/L) (CLSI, 2017). Categorization of strains as S/I/R referred to the current CLSI breakpoints. Screening of carbapenemase-producing strains was performed by using the I-C4000 test, previously shown to have excellent sensitivity and specificity (Fournier et al., 2013a). Briefly, a disk of imipenem (10 μg load) and a disk of imipenem plus 4,000 μg of cloxacillin were deposited on to the surface of a Mueller-Hinton agar plate previously inoculated with a bacterial suspension at 0.5 McFarland. After 18 h of incubation at 37°C, a difference >5 mm between the inhibition diameters around the two disks—due to inhibition of natural ß-lactamase AmpC by cloxacillin, was indicative of absence of carbapenemase production.

### Genotyping experiments

The clonal relatedness of strains was investigated by both MLST (MultiLocus Sequence Typing) and MLVA (Multiple-Locus Variable-number tandem-repeat Analysis) targeting 10 VNTR (Variable Number Tandem Repeats), as previously reported (Vu-Thien et al., 2007; van Mansfeld et al., 2009). The allelic profile of an isolate was expressed as the number of repeats for each VNTR in the following order: ms142, ms211, ms212, ms213, ms214, ms215, ms216, ms217, ms222, and ms223. A difference of one repeat between two strains for any of the VNTR was considered as discriminant.

### Quantitative RT-PCR

The transcripts of genes *copR, czcC, czcR, mexB, mexE, mexY, oprD*, and PA1797 were quantified in a Rotor gene RG6000 apparatus (Qiagen) with fluorescent intercalating dye SybrGreen (Fast SybrGreen kit, Qiagen). The primers used for RT-qPCR are listed in Table S1. Briefly, RNA was extracted en masse from mid-log phase cultures (*A*_600 nm_ equal to 0.8) in MH broth with the RNeasy Plus kit (Qiagen). DNA was removed by RNase-free DNAse treatment, and 2 μg of purified RNA were incubated with ImpromII reverse transcriptase according to the manufacturer's recommendations (Promega). The transcript levels were relativized to the PAO1 values after internal gene normalization (*uvrD*), as previously described (Jo et al., 2003). Data presented are means of four determinations from two independent experiments.

### DNA amplification and sequencing

Potential mutations in gene *oprD* and its promoter region were searched in the tested isolates. The PCR amplicons were sequenced on both strands by using the BigDye Terminator V3.1 cycle sequencing kit (Applied Biosystems) and specific primers (Table S1), in an RUO3500 Genetic Analyzer (Applied Biosystems). Genes *mexR, nalC, nalD, mexZ, parS, parR, mexS, copS, copR*, and/or *czcR* were sequenced in strains showing significant overexpression of *mexB, mexY, mexE, copR*, and/or *czcC* genes. The resultant nucleotide and amino acid sequences were aligned with that of reference strains PAO1 (BenBank, accession no. NP249649), PA14 (accession no. CP000438), LESB58 (accession no. WP003107743), and PA7 (accession no. WP012076895), deposited in GenBank (https://blast.ncbi.nlm.nih.gov).

## Results

### Features of 30 animal isolates of *P. aeruginosa* non-susceptible to carbapenems

Carbapenems are not licensed for the treatment of animals. However, 30 imipenem and/or meropenem non-susceptible *P. aeruginosa* were retrospectively identified in a collection of 527 veterinary strains gathered between 2008 and 2014 by the French network Resapath (Table 1). These resistant strains had been the cause of otitis in dogs (*n* = 13), and pulmonary infections in cats, dogs, and a bovine (*n* = 6). Their clonal relatedness was explored by MLVA, which allowed the identification of 25 different genotypes. Most were singletons, but 3 clones included 2 (clones A, G) and 4 (clone B) isolates, respectively. Suggesting that some strains may be more transmissible than others, analysis of the Resapath database indicated that clones A and G had infected animals from distant geographical regions in France (Moselle, Val-de-Marne, and two distinct towns in the district of Alpes Maritimes, respectively). Interestingly, clone B strains were isolated from unrelated animals admitted to the same veterinary clinic in 2010 (three strains between July and October) and in 2014 (one strain in December), probably as part of an outbreak. MLST genotyping experiments showed that clone B belongs to international high-risk complex ST395, known for its propensity to spread in hospitals and to develop multidrug resistance (Libisch et al., 2009; Fernandez-Olmos et al., 2013; Martin et al., 2013; Valot et al., 2014). ST395 can also be encountered in the natural environment as well as in waste waters (Slekovec et al., 2012; Teixeira et al., 2016). Furthermore, we found that 21 strains of the collection (70%) belonged to various sequence types initially identified among clinical isolates (Table 1) (Samuelsen et al., 2010; Garcia-Castillo et al., 2011; Cho et al., 2013; Gomila et al., 2013; Perez et al., 2014). In apparent contradiction with previous studies, our data thus indicated that clones ST395 and ST233 are able to infect humans and animals (Wiehlmann et al., 2007; Kidd et al., 2012; Haenni et al., 2015).

**Table 1:** Features of the 30 carbapenem-non-susceptible veterinary strains of *P. aeruginosa*.

**Strains**	**Animal**	**Origin**	**Sequence type (ST)^a^**	**MLVA group**	**MIC^b^ (mg/L)**		**Porin OprD**	
					**IPM**	**MPM**	**Amino acid sequence**	**Gene expression^c^**
24362	Bovine	Respiratory	**683**	A	**4**	2	wt^d^	−**4.13**
25306	Cat	Respiratory	**395**	B	**16**	**4**	Δ_nt 410−420_^e^	nd
25333	Cat	Urine	**233**	C	**4**	2	wt^d^	−**2.44**
25334	Cat	Respiratory	**564**	D	**8**	2	G_404_C^d^	−**3.33**
25356	Dog	Ear	**385**	E	**4**	1	wt^d^	−**2.38**
25380	Dog	Ear	663	F	2	**4**	wt^d^	−1.10
25401	Dog	Pus	871	G	2	**4**	wt^f^	−1.76
25572	Cat	Ear	871	G	2	**4**	wt^f^	−1.80
25747	Dog	Respiratory	**395**	B	**16**	**4**	Δ_nt 410−420_^e^	nd
25752	Dog	–	**560**	H	**8**	2	S_57_E, S_59_R^g^	−**7.69**
25827	Dog	–	**395**	I	2	**4**	wt^d^	−1.06
25828	Cat	Respiratory	**395**	B	**16**	**4**	Δ_nt 410–420_^e^	nd
26103	Dog	Ear	2,502	J	**8**	**4**	wt^h^	−**2.00**
26276	Dog	Urine	**395**	B	**16**	**4**	Δ_nt 410–420_^e^	nd
26292	Dog	Ear	**612**	K	1	**4**	wt^d^	−1.26
26427	Dog	Ear	**683**	A	**4**	2	wt^d^	−**3.62**
26860	Dog	Respiratory	884	L	**8**	**8**	wt^f^	−**9.68**
26877	Dog	–	**281**	M	**8**	2	wt^d^	−**16.88**
27636	Dog	Ear	**253**	N	**8**	**4**	IS*Pst3*^e^	nd
30124	Dog	Ear	480	O	**4**	**4**	wt^d^	−**7.02**
35941	Dog	–	**17**	P	**4**	2	wt^d^	−**2.49**
36140	Dog	Abscess	**348**	Q	2	**4**	wt^f^	−1.18
36145	Dog	Ear	**244**	R	2	**4**	wt^h^	1.7
36150	Dog	Ear	2,503	S	**4**	0.5	S_57_E, S_59_R^g^	−**2.03**
36163	Dog	Ear	**267**	T	2	**4**	wt^h^	2.32
36171	Dog	Ear	**471**	U	2	**4**	wt^h^	1.92
37241	Dog	–	**309**	V	**16**	**4**	IS*Pa1328*^e^	nd
37248	Dog	Cutaneous	**253**	W	2	**4**	wt^f^	1.27
37249	Dog	Ear	2,504	X	**16**	**16**	wt^d^	−**9.67**
37257	Dog	Ear	2,505	Y	**4**	2	wt^d^	−**2.50**

a *ST previously associated with P. aeruginosa strains of human origin are indicated in boldface*.

b *Non-susceptible (intermediate and resistant) strains according to CLSI breakpoints, are indicated in boldface*.

c *Relative to expression in strain PAO1. Values are means from two independent experiments, each including duplicate determinations. Gene oprD is considered as significantly down-regulated (in boldface) when its expression is at least 2-fold less than those in PAO1*.

d *LESB58-like sequence*.

e *Absence of porin production because of gene oprD disruption*.

f *PA14-like sequence*.

g *PP2-like sequence*.

h *PAO1-like sequence*.

*IMP, imipenem; MPM, meropenem; –, unknown; nd, not determined; wt, wild-type sequence*.

As mentioned in Table 1, only 10 strains (33.3%) were intermediate (I) or resistant (R) to both imipenem and meropenem with respect to the CLSI breakpoints. Some strains were also I or R to major antipseudomonal antibiotics such as cefepime (6.6%), piperacillin/tazobactam (6.6%), ticarcillin (83.3%), ciprofloxacin (40%), and aztreonam (36.6%), but all were susceptible (S) to ceftazidime, tobramycin, amikacin, and colistin (Table 2). According to an international consensus, 12 (40%) strains fitted with the definition of multi-drug resistant bacteria (MDR) (Magiorakos et al., 2012).

**Table 2:** Distribution of the carbapenem-non-susceptible veterinary strains of *P. aeruginosa* according to drug MICs.

**Antibiotic**	**Number of strains at indicated MIC (mg/L)**											
	**0.12**	**0.25**	**0.5**	**1**	**2**	**4**	**8**	**16**	**32**	**64**	**128**	**256**
Imipenem					9	9^a^	**6**^b^	**6**				
Meropenem			2	1	8	17	**1**	**1**				
Ceftazidime				4	15	8	3					
Cefepime				3	2	9	14	2				
Aztreonam					1	10	8	5	**6**			
Ticarcillin								5	12	9	3	**1**
Piperacillin/tazobactam					1	10	10	7	2			
Tobramycin			8	15	7							
Amikacin				1	11	3	6	9				
Ciprofloxacin	3	5	7	3	5	**5**	**2**					
Colistin			2	24	4							

a *Shaded areas correspond to intermediate and resistant strains*.

b *Resistant strains are indicated in boldface*.

### Alterations of porin OprD

Screening of carbapenemase production with IC-4000 test was negative for all the veterinary strains. As the loss of specific porin OprD is the main cause of carbapenem resistance in human isolates of *P. aeruginosa* (Lister et al., 2009; Fournier et al., 2013b), we analyzed the sequence of gene *oprD* and its promoter region among the selected strains. In the four clone B strains belonging to ST395, *oprD* was disrupted by a same deletion of 11 nucleotides, while in other two strains the gene was interrupted by an insertion sequence, IS*Pa1328* or IS*Pst3* (Table 1). Of note, IS*Pa1328* has previously been identified in carbapenem-resistant hospital strains in France, United-States, Lebanon, and Japan (Wolter et al., 2004; Sanbongi et al., 2009; Fournier et al., 2013b; Al Bayssari et al., 2015).

The primary sequence of porin OprD is known to be highly variable in clinical and environmental *P. aeruginosa*, including those susceptible to carbapenems (Ocampo-Sosa et al., 2012). Most of the veterinary strains exhibited OprD sequences identical to that of reference strain LESB58 (*n* = 12), PA14 (*n* = 5), or PAO1 (*n* = 4). A so far uncharacterized G_404_C substitution was identified in one strain, 25334, producing a LESB58-like porin. Finally, the last two strains, 25752 and 36150, were found to harbor the same S_57_E and S_59_R changes in a PP2-like OprD. According to Ocampo-Sosa A. et al., these variations, which are located in porin loop L1 would not impact the diffusion of carbapenems through the outer membrane, suggesting that 25752 and 36150 resist to carbapenems via a mechanism other than membrane impermeability (Ocampo-Sosa et al., 2012).

### Overexpression of efflux pumps and down-regulation of OprD

In *P. aeruginosa*, the intracellular penetration of carbapenems may be impaired consecutively to mutational loss of porin OprD (as reported above), mutation-driven changes in the OprD structure, or the presence of reduced amounts of OprD in the outer membrane (Fournier et al., 2013b). Indeed, some mutations in the promoter region of *oprD* or in regulatory genes that control efflux pumps such as CzcCBA, MexEF-OprN, and MexXY negatively influence *oprD* transcription, leading to a 2- to 8-fold increase in imipenem MIC (Lister et al., 2009; Muller et al., 2011; Fournier et al., 2013b). RT-qPCR experiments demonstrated that 14 veterinary strains including isolates 25334, 25752, and 36150 that exhibit novel OprD variants, expressed the *oprD* gene 2- to 16.8-fold less than wild-type reference strain PAO1 (Table 1). None of these isolates harbored mutations in the promoter region of *oprD*. On the other hand, three of them (25752, 36150, and 37249) significantly overexpressed gene *czcC* (3.25-, 4.90-, and 17.27-fold more than PAO1, respectively). The efflux system CzcCBA contributes to the intrinsic resistance of *P. aeruginosa* to zinc and cadmium ions, and is under the positive control of two-component regulatory systems CzcRS, and CopRS (Hassan et al., 1999; Okamoto et al., 2002; Caille et al., 2007). Phosphorylation of response regulators CzcR and CoprR by histidine kinases CzcS and CopS, respectively results in activation of operon *czcCBA* and concomitant down-regulation of gene *oprD* (Caille et al., 2007). In agreement with this, sequence analysis of loci *czcRS* and *copRS* demonstrated the occurrence of amino acid substitutions in sensor protein CzcS from strains 25752 (A_110_Y, S_468_A), 36150, (S_468_A), and 37249 (P_226_D), and in CopS from strains 25752 (A_304_Y) and 37249 (L_258_R) compared with PAO1, pointing to a possible cause of activation of CzcS, CopS, and *in fine* CzcCBA. Supporting this interpretation, expression of genes *czcR* and *copR* in the three strains was found to be 3.11- to 7.42-fold higher than in PAO1. Both genes are known to be positively controlled by their respective sensors at the transcriptional level (Okamoto et al., 2002). Mutational alteration of two component system(s) CzcRS and/or CopRS is thus clearly associated with carbapenem resistance in both clinical and animal strains of *P. aeruginosa* (Fournier et al., 2013b).

Four strains showing a downregulated *oprD* gene (namely, 25334, 26103, 26860, and 37249) appeared to have *mexE* transcripts 89.5- to 3,057-fold more abundant than in PAO1. As demonstrated previously, overproduction of efflux system MexEF-OprN in clinical *nfxC* strains mainly results from mutations inactivating or impairing the activity of putative oxidoreductase MexS (Richardot et al., 2016). Analysis of the four veterinary *mexE*-overexpressing strains indeed revealed disruption of *mexS* in three of them (del_20nt_ in isolates 25334 and 37249, lack of amplification of *mexS* gene probably due to an IS in 26103 and a M_227_V substitution in the enzyme from 26860). To the best of our knowledge, disruption of *mexS* by an insertion sequence has never been reported so far in clinical or *in vitro-*selected *nfxC* mutants. The same also applies to the M_227_V change observed in strain 26860, though its contribution to the MexS-dependent activation of global regulator MexT, with subsequent upregulation of efflux MexEF-OprN and downregulation of porin OprD, remains to be confirmed. All the veterinary *nfxC* mutants exhibited a significant resistance to imipenem (MIC = 8 mg/L). Of interest, the concomitant dysregulation of systems CzcCBA and MexEF-OprN in strain 37249 had cumulative effects on imipenem MIC (16 mg/L) (Table 1).

**Figure 1:**
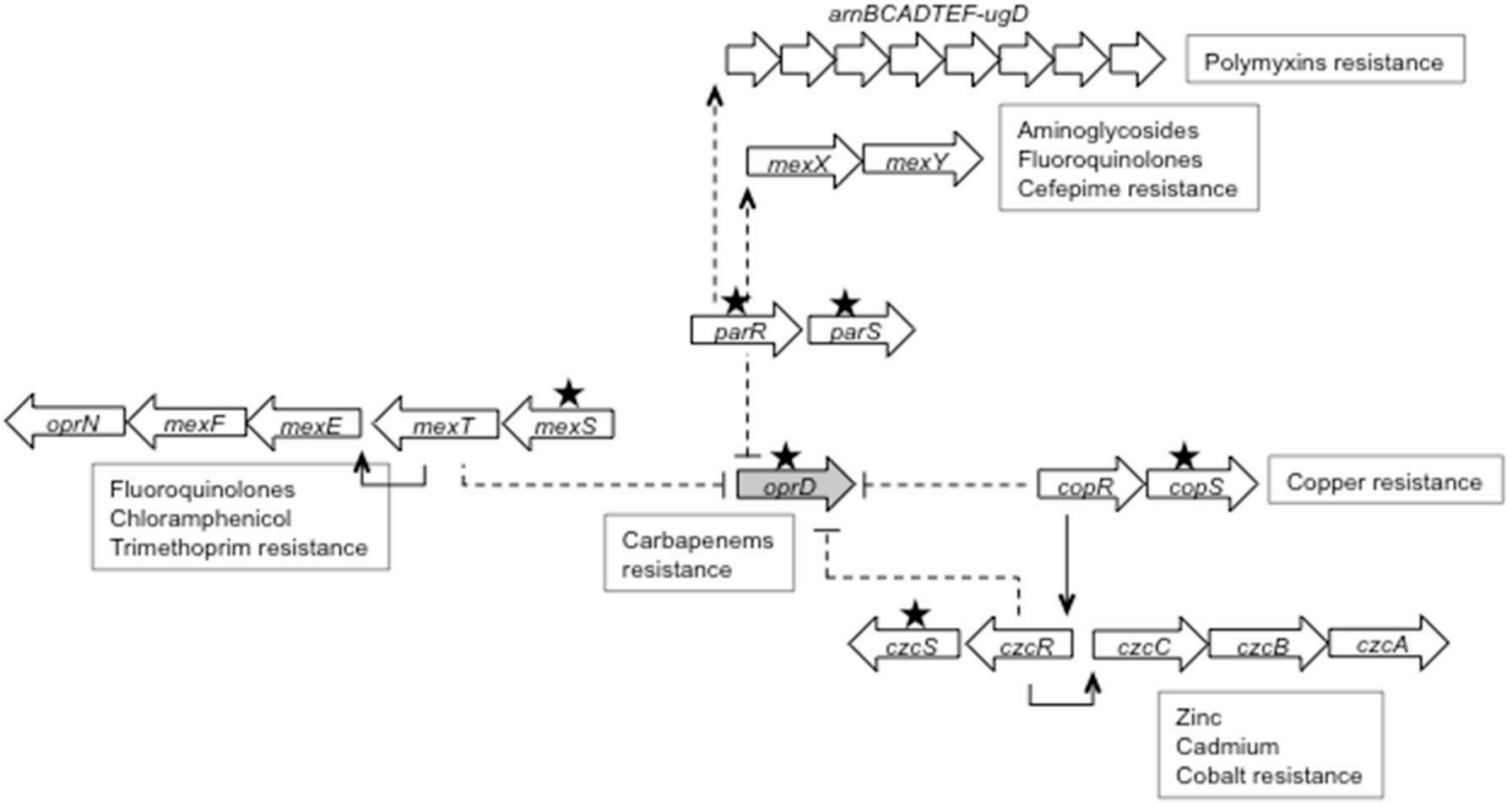
Schematic representation of genes involved in down-regulation of gene *oprD* in veterinary *P. aeruginosa* strains. The *oprD* gene is repressed by mutations (represented by black stars) activating the two-component systems ParRS (in genes *parR, parS*), CopRS (in gene *copS*) and CzcRS (in gene *czcS*), respectively. While induction of operon *mexXY* expression by ParRS leads to a higher resistance to aminoglycosides, fluoroquinolones and cefepime, activation of CopRS or CzcRS results in an increased resistance to metal ions. In addition, ParRS-dependent positive control of LPS modification operon *arnBCADTEF-ugd* is responsible for a low resistance to polymyxins. Defective porin OprD production and carbapenem resistance may also arise when the LysR-type regulator MexT is activated following alteration of the gene encoding putative oxidoreductase MexS. Gene *mexS* mutants are characterized by an increased resistance to fluoroquinolones, trimethoprim, and chloramphenicol as a result of operon *mexEF-oprN* overexpression.

Finally, repression of gene *oprD* may be caused by mutational activation of ParRS, a two-component system, which positively controls operon *mexXY* and an adjacent gene of unknown function, PA1797 (Muller et al., 2011). As indicated in Table 3, among the 14 strains exhibiting low *oprD* mRNA levels, eight significantly upregulated genes *mexY* (from 9.44- to 72.57-fold) and PA1797 (from 7.50- to 95.35-fold), compared with PAO1. Amino acid substitutions were found in response regulator ParR (strain 35941) and sensor ParS (*n* = 7 strains) (Table 3). None of these except A_138_T in ParS has been reported so far in *agrW2* mutants isolated in the clinical setting (Muller et al., 2011; Guénard et al., 2014). They are located in the periplasmic (L_50_P), the HAMP (Histidine kinase, Adenylyl cyclase, Methyl-accepting chemotaxis proteins and phosphatases) (R_185_G), the HisKA (A_215_T), and the ATPase (A_324_V) domains of ParS. The finding that ParRS-controlled genes *mexXY* and PA1797 are overexpressed in those strains strongly supports the notion of a constitutive activation of ParRS by such mutations, as observed in clinical *P. aeruginosa* isolates (Muller et al., 2011; Guénard et al., 2014). Providing further evidence of ParRS activation, colistin MIC was 2-fold higher for the eight *agrW2* mutants than for the rest of the collection. Indeed, in addition to downregulating the *oprD* gene, ParRS is able to upregulate expression of LPS modification operon *arnBCADTEF-ugd* in this type of mutants, with consecutive increase in resistance to polymyxins (Muller et al., 2011). Figure 1 depicts the regulatory pathways involved in repression of *oprD* gene in the veterinary strains studied.

**Table 3:** Characterization of ParRS-dependent MexXY overproducing mutants among the 14 strains exhibiting low *oprD* mRNA levels.

**Strains**	**Gene expression^a^**		**ParRS primary sequence**	
	***mexY***	**PA1797**	**ParR**	**ParS**
24362	**72.57**	**7.50**	wt^b^	R_185_G^c^
25333	**16.77**	**95.35**	wt^b^	A_215_T^c^
25334	0.73	nd	nd	nd
25356	**33.05**	**35.02**	wt^b^	A_215_T^c^
25752	1.09	nd	nd	nd
26103	3.40	1.10	wt^b^	wt^c^
26427	**40.17**	**11.60**	wt^b^	R_185_G^c^
26860	1.74	nd	nd	nd
26877	**9.44**	**47.60**	wt^b^	A_138_T^c^
30124	**21.71**	**15.40**	wt^b^	A_324_V^c^
35941	**23.37**	**5.70**	L_11_F^b^	wt^c^
36150	2.74	nd	nd	wt^c^
37249	1.18	nd	nd	wt^c^
37257	**49.76**	**23.90**	wt^b^	L_50_P^c^

a *Relative to expression in strain PAO1. Values are means from two independent experiments, each including duplicate determinations. Genes mexY and PA1797 are considered as significantly up-regulated (in boldface) when their respective expression is at least four-fold higher than those in PAO1*.

b *PAO1-like sequence*.

c *PA14- and LESB58-like sequence*.

*nd, not determined; wt, wild-type sequence*.

### Resistance to meropenem correlates with *mexAB-oprM* overexpression

When overproduced, the efflux system MexAB-OprM increases the resistance of *P. aeruginosa* to ß-lactams including meropenem and doripenem, but not to imipenem (Masuda et al., 2000; Okamoto et al., 2002). To assess the role of this pump in the meropenem resistance of veterinary strains, gene *mexB* transcripts were quantified in all the isolates by RT-qPCR. As indicated in Table 4, 12 out 30 strains (40%) appeared to overexpress *mexB* from 3.4- to 15.75-fold, and to be 4- to 16-fold more resistant than wild-type strain PAO1 to meropenem (MIC of meropenen equal to 0.5 mg/L in PAO1) (Table 1). Transcription of *mexAB-oprM* is subject to a complex regulation involving two repressors, NalD and MexR, which respectively bind to a proximal and a distal promoter upstream of the operon (Poole et al., 1996; Morita et al., 2006). Recently, it was reported that a response regulator, named CpxR, is also able to activate *mexAB-oprM* via its interaction with the distal promoter mentioned above, in *mexR* knock-out mutants (Tian et al., 2016) Finally, another regulator, NalC, indirectly influences *mexAB-oprM* transcription by modulating the expression of a two-gene operon in which *armR* encodes an anti-MexR protein (Cao et al., 2004; Daigle et al., 2007). Therefore, many genetic events leading to the inactivation or alteration of protein MexR (*nalB* mutants), NalC (*nalC* mutants), or NalD (*nalD* mutants) are expected to upregulate the *mexAB-oprM* operon, and so to increase meropenem MIC in clinical isolates (Ziha-Zarifi et al., 1999; Higgins et al., 2003; Cao et al., 2004; Llanes et al., 2004; Sobel et al., 2005a; Chalhoub et al., 2016). Sequencing of the corresponding genes in the 12 MexAB-OprM-overproducers identified two isolates containing disrupted *mexR* genes (deletion of 1 and 10 nucleotides in 26292 and 36140, respectively), and a third one (36171) encoding a L_54_P substitution in the DNA binding domain of MexR (Table 4) (Lim et al., 2002). Four other strains were found to carry mutations generating a frameshift in gene *nalC* (deletion of 12 nucleotides in 26860 and 36145), a truncated NalC (R_143_stop in 25827), or amino acid substitutions in this repressor (E_153_D, A_186_D in 25333). Five remaining strains harbored disruptive mutations in *nalD* (strains 25401, 25572, 25333, 25380, 36163) while in the strain 37248 the gene coding sequence was interrupted by an IS*As2* element (Table 4). Reminiscent of this, *mexR* was reported to be inactivated by another insertion sequence, IS*21*, in a MexAB-OprM upregulating clinical strain (Boutoille et al., 2004). As already observed in clinical strains (Ziha-Zarifi et al., 1999; Srikumar et al., 2000; Llanes et al., 2004; Sobel et al., 2005a), mutations in both *nalC* and *nalD* were present in one strain, 25333, though with no apparent influence on *mexB* expression and/or meropenem MIC, as compared with single mutants. To our knowledge, all the *nalB* (*n* = 3), *nalC* (*n* = 3), *nalD* (*n* = 5), and *nalC/D* (*n* = 1) mutations reported here are novel.

**Table 4:** Characterization of 12 MexAB-OprM overproducing mutants.

**Strains**	***mexB* expression^a^**	**Primary sequence of MexAB-OprM regulators**		
		**MexR**	**NalC**	**NalD**
25333	6.60	wt^b^	E_153_D, A_186_T^b^	Q_134–_stop
25380	11.48	wt^b^	wt^c^	W_49–_stop
25401	4.95	wt^c^	wt^d^	Δ_nt 460–71_^e^
25572	3.40	wt^c^	wt^d^	Δ_nt 460–71_^e^
25827	7.95	wt^c^	R_143–_stop^d^	wt^b^
26292	8.94	Δ_nt 205_^e^	wt^c^	wt^b^
26860	6.23	wt^b^	Δ_nt 37–48_^e^	wt^b^
36140	15.72	Δ_nt 55–64_^e^	wt^c^	wt^b^
36145	13.48	wt^b^	Δ_nt 37–48_^e^	wt^b^
36163	12.55	wt^b^	wt^b^	Δ_nt 461–70_^e^
36171	15.75	L_54_P	wt^c^	wt^b^
37248	8.03	wt^c^	wt^c^	IS

a *Relative to expression in strain PAO1. Values are means from two independent experiments, each including duplicate determinations. Gene mexB is considered as significantly overexpressed when the expression levels are at leastthree-fold higher than those in PAO1.– not determined*.

b *PAO1-like sequence*.

c *PA14-like sequence*.

d *LESB58-like sequence*.

e *Nucleotide deletions or IS inactivating mexR, nalB, or nalD genes*.

## Discussion

Carbapenems are critically-important antibiotics, restricted to human use. In this study, we show that carbapenem resistance may emerge in animal strains of *P. aeruginosa* mainly as a collateral consequence of efflux pump dysregulation. Since susceptibility to these ß-lactams is usually not assessed by veterinary laboratories, resistance in animal strains is often ignored or underestimated (Kroemer et al., 2014; Ludwig et al., 2016). Non-susceptibility rates to meropenem or/and imipenem were as high as 5.7% (30 out 527) in the Resapath collection of veterinary *P. aeruginosa* isolates, confirming the occurence of carbapenem resistance in strains infecting animals (Rubin et al., 2008; Lin et al., 2012). Though metallo-ß-lactamase VIM-2 producing *P. aeruginosa* were recovered from fowl and cattle in Lebanon (Al Bayssari et al., 2015), none of the strains of our collection happened to express a carbapenemase. Instead, their reduced susceptibility to carbapenems resulted from a variety of non-enzymatic mechanisms. Interestingly, complete suppression of the OprD porin itself by mutations or IS did not represent a major cause of resistance to these agents in animal strains, compared with their human counterparts. All six strains with disrupted *oprD* genes belonged to genotypes found in humans, such as ST395. In contrast to other findings (Haenni et al., 2015), the 24 remaining veterinary *P. aeruginosa* of the study were distributed into 22 genotypes most of which (*n* = 14) have initially been described in the hospital setting. Since inactivation of gene *oprD* is the cause of a very specific resistance to carbapenems, and that carbapenems are not used in veterinary medicine in France, our hypothesis is that the six OprD null mutants characterized here have been selected under carbapenem therapy in humans rather than by other drugs in animals. If this assumption is correct, those strains would have been transmitted from humans to animals. On the other hand, porin OprD showed the same primary structure as those of strain PAO1, PA14, LESB58, or PP2 in 21 veterinary isolates, or to carry amino acid variations of doubtful significance in three others. In all these bacteria, gene *oprD* expression was repressed by mutations occurring in several genes known to regulate active efflux pumps such as MexAB-OprM (*n* = 12), MexEF-OprN (*n* = 4), MexXY (*n* = 8), and CzcCBA (*n* = 3). Overexpression of operon *mexAB-oprM* was related to mutations or IS in genes *mexR* (*nalB* mutants, *n* = 3), *nalC* (*n* = 4), and/or *nalD* (*n* = 6). To our knowledge, *nalC* and *nalD* mutants have never been described in veterinary strains. In year 2001, 12 MexAB-OprM, MexEF-OprN, and/or MexXY overproducers were identified among multidrug resistant *P. aeruginosa* from canine ears, essentially (Beinlich et al., 2001). However, the susceptibility of these bacteria to carbapenems was not assessed, and the search of mutations was restricted to genes *mexR* and *mexZ*.

As shown in this work, operons *mexEF-oprN, mexXY*, and *czcCBA* were overexpressed in the selected isolates as a result of mutations impairing the activity of oxidoreductase MexS, and mutations activating signal transducers ParRS, and CopS/CzcS, respectively. Our study confirms the contribution of pump MexXY in aminoglycoside resistance of strains from dogs and cats (Chuanchuen et al., 2008; Poonsuk and Chuanchuen, 2012). Interestingly, some of the MexXY overproducers mentioned previously harbored wild-type genes *mexZ*, thus suggesting a possible alteration of two-component system ParRS as cause of *mexXY* dysregulation and carbapenem resistance. ParRS (*agrW2*) mutants accounted for 26.7% (eight out 30) of the carbapenem non-susceptible strains of our collection. Consistent with other data from our lab (Muller et al., 2011), these strains were also more resistant to aminoglycosides, fluoroquinolones, and polymyxins than wild-type control PAO1.

As in the hospital setting, (Jalal et al., 2000; Pumbwe and Piddock, 2000; Llanes et al., 2004) *P. aeruginosa* strains turned out to overproduce two active efflux pumps simultaneously, including the novel combination CzcCBA plus MexEF-OprN. Co-expression of two efflux systems in strains 26860 (MexAB-OprM plus MexEF-OprN) and 37249 (MexEF-OprN plus CzcCBA) was associated with relatively high meropenem MICs (8 and 16 mg/L, respectively), as compared with single pump overproducers (2–4 mg/L), indicating that gain-of-efflux mutations may have cumulative effects on the resistance to this major antibiotic. Conversely, meropenem resistance in strain 25333 (MexAB-OprM plus MexXY) was low (2 mg/L), perhaps because of opposite effects of the mutations.

## Conclusion

Overall, this study demonstrates the occurence of carbapenem resistance in animal strains of *P. aeruginosa* mainly as a result of mutational alteration of genes controlling efflux pumps. Selection of such mutants could be explained by the use of disinfectants and antibiotics in veterinary practice (Heuer et al., 2005). Empirical and curative therapies based on topical application of a fluoroquinolone (enrofloxacin, marbofloxacin) or an aminoglycoside (gentamicin, amikacin) are common to eradicate *P. aeruginosa* in otitis media or external otitis in companion animals such as dogs and cats (De Briyne et al., 2014). Fluoroquinolones are prone to select gain-of-efflux mutants in *P. aeruginosa* (Le Thomas et al., 2001; Morero et al., 2011; Riou et al., 2016). Though our results suggest that mutants with disrupted *oprD* genes might be transmitted from humans to animals, the presence in animals of regulatory mutants combining a membrane impermeability and efflux mechanisms constitutes a potential risk for fragile outpatients such as those with cystic fibrosis, to be contaminated by multidrug resistant strains.

## Author contributions

MH, PP, JM, and KJ contributed to the conception of the study; MB, PC, and MH performed the data analyses; PP, and KJ wrote the manuscript; MH, and JM helped perform the analysis with constructive discussions.

### Conflict of interest statement

The authors declare that the research was conducted in the absence of any commercial or financial relationships that could be construed as a potential conflict of interest.
