# Supplementary material for: Resistance of Animal Strains of Pseudomonas aeruginosa to Carbapenems
Source: Front Microbiol. 2017 Sep 29;8:1847. doi: 10.3389/fmicb.2017.01847 (PMC5626926; doi:10.3389/fmicb.2017.01847)
Supplement: Supplementary file 1 [file Table1.DOCX]

**Supplemental data.**

**Table S1.** Primers used in the study.

| Primer | | Sequence (5'–3') | Reférence | |
| --- | --- | --- | --- | --- |
| *Oligonucleotides for amplification/Sequencing* | | | | |
| sqpromD1 | | CAATTTGTGCACGGAGTTTG | | (Fournier *et al.*, 2013b) |
| OprD2 | | GGGGTGTTTTCGCAGAGTAA | | (Fournier *et al.*, 2013b) |
| OprD1 | | CCAAGAAACACTGCGTGCTA | | (Fournier *et al.*, 2013b) |
| OprDseq1 | | CCTGCGTAGGTGGCATAGAG | | (Fournier *et al.*, 2013b) |
| OprDseq2 | | TCCAAGACCATGCTGAAGTG | | (Fournier *et al.*, 2013b) |
| Seq-mexS-1 | | GAACAGGATCAGCAGGTTCA | | (Richardot *et al.*, 2016) |
| Seq-mexS-2 | | CCACCGGGGTGAGTACCT | | (Richardot *et al.*, 2016) |
| Seq-mexS-3 | | GTCTCGGCTTCGAACTGG | | (Richardot *et al.*, 2016) |
| Seq-mexS-4 | | GGTGAAATCCATCAGGCAGT | | (Richardot *et al.*, 2016) |
| Seq-mexS-5 | | GCAAGCTGGTGCTGTATGG | | (Richardot *et al.*, 2016) |
| Seq-mexS-6 | | GAAGGCGACTTCGTCTGG | | (Richardot *et al.*, 2016) |
| seqMexR S1 | | CATGGCCCATATTCAGAACC | | This study |
| seqMexR S2 | | AGGTTTCTTCCCTCCAGCTC | | This study |
| seqMexR S3 | | CGACGTCCATGTATTGAAGC | | This study |
| seqMexR S4 | | GCGGATACCTGAAACGAAAA | | This study |
| NalCfw1 | | CCTGGAGGCTTGAACGGA | | This study |
| NalCrv1 | | AGATCCACCTCACCGAACTG | | This study |
| NalCseqfw1 | | CAGAGCCTCTATCGCCTGG | | This study |
| NalCseqrv1 | | GCATCTCGAGAAACTGGCAG | | This study |
| NalDfw | | GAGACCGATTCGACTACCCA | | This study |
| NalDrv | | AGTTTTCAAGGTTCAGGGCA | | This study |
| seqPA1799A1 | | GGTCGACCACGAAGATCG | | (Muller *et al.*, 2011) |
| seqPA1799A2 | | GCATATAATGCCAGCCGATT | | (Muller *et al.*, 2011) |
| seqPA1798C1 | | GCCAGGCAGGGGAAATACT | | (Muller *et al.*, 2011) |
| seqPA1798C2 | | CATACCAGCAGGGCGGATG | | (Muller *et al.*, 2011) |
| seqPA1798C3 | | AAGAACCTGCTGGTGGTACG | | (Muller *et al.*, 2011) |
| seqPA1798C4 | | ATGCGGATCTGTTCGACCT | | (Muller *et al.*, 2011) |
| seqPA1798C5 | | CGAACTGGAGGAAATGGTCT | | (Muller *et al.*, 2011) |
| seqPA1798C6 | | GAAAGATGCATTGCACGAAA | | (Muller *et al.*, 2011) |
| seqZ1 | | GCAGCCCAGCAGGAATAG | | (Muller *et al.*, 2011) |
| seqZ2 | | GCCTGTCGGTGCTCTACATC | | (Muller *et al.*, 2011) |
| CopR-F | | GTTACCCACTGATTACGATGCT | | This study |
| CopR-R | | TGCTGAAGATCAATCCGGC | | This study |
| CopS-F | | AGCGCCTGATCCATACCGTG | | This study |
| CopS-R | | GAAACGCTCGAAGGCATGAAA | | This study |
| seqCopSfw1 | | CTGGAGGACTATCGCGAGG | | This study |
| seqCopSrv1 | | TAGAACTCCAGCAGCGAGTC | | This study |
| CzcR-F | | GCAGCGCAACGATATGAAATT | | This study |
| CzcR-R | | CAATTGCAGGTTTTCCCGCT | | This study |
| CzcS-F | | AAGATCGACGACGGCTTCGA | | This study |
| CzcS-R | | CTGTTCCTCGCCGGTTTCT | | This study |
| seqCzcSfw1 | | CCCTTCCTCCTGCCATTGAT | | This study |
| seqCzcSrv1 | | CGAGCATGTGGTTGAAGCTG | | This study |
| *RT-qPCR primers* | | | |  |
| UvrD1 | CACGCCTCGCCCTACAGCA | | (Jo *et al.*, 2003) |  |
| UvrD2 | GGATCTGGAAGTTCTCGCTCAGC | | (Jo *et al.*, 2003) |  |
| OprD1 | ATCTACCGCACAAACGATGAAGG | | (Dumas *et al.*, 2006) |  |
| OprD2 | GCCGAAGCCGATATAATCAAACG | | (Dumas *et al.*, 2006) |  |
| MexE1 | CCAGGACCAGCACGAACTTCTTGC | | (Dumas *et al.*, 2006) |  |
| MexE2 | CGACAACGCCAAGGGCGAGTTCACC | | (Dumas *et al.*, 2006) |  |
| MexB1 | ATCCGCCAGACCATCGCCA | | (Hocquet *et al.*, 2006) |  |
| MexB2 | CATCACCAGGAACACGAGGAGG | | (Hocquet *et al.*, 2006) |  |
| MexY1A | TTACCTCCTCCAGCGGC | | (Jeannot *et al.*, 2005) |  |
| MexY1B | GTGAGGCGGGCGTTGTG | | (Jeannot *et al.*, 2005) |  |
| RTPA5471Fw | GCATCGAAGCCCTGGATCTA | | This study |  |
| RTPA5471Rv | GCAGGCTGACATGCTCTTG | | This study |  |
| PA1797RTC1 | GGACCCTTTGCAGATGACTC | | (Muller *et al.*, 2011) |  |
| PA1797RTC2 | CGGAGTGTTTCCTGAGAAGC | | (Muller *et al.*, 2011) |  |
| RTMexC3 | GTACCGGCGTCATGCAGGGTTC | | (Dumas *et al.*, 2006) |  |
| RTMexC4 | TTACTGTTGCGGCGCAGGT | | (Dumas *et al.*, 2006) |  |
| CopR L | ACCTCGAACTCGATTTGCTG | | This study |  |
| CopR R | TCGCTGTCGAAGTTCATGTC | | This study |  |
| CopS L | GTTCGACCGGTTCTATCGTG | | This study |  |
| CopS R | AGCGAAATCGATGACGAAAC | | This study |  |
| CzcC1 | GGTCAGCATCGGCAGCAAGTACG | | This study |  |
| CzcC2 | GGTCGTAGGCCTGTACCGCTTCG | | This study |  |
| CzcS1 | TACGCCAGCTCTCGCAGTTCTCC | | This study |  |
| CzcS2 | TGTCCACCTGCACCAGGAACAGC | | This study |  |
| CzcR1 | GTCATCACCCGGACGCAGATCAT | | This study |  |
| CzcR2 | GTAGCCCGACGCCGCGAATGGTAT | | This study |  |
